# Supplementary material for: Measuring financial risk protection in health benefits packages: scoping review protocol to inform allocative efficiency studies
Source: BMJ Open. 2019 May 28;9(5):e026554. doi: 10.1136/bmjopen-2018-026554 (PMC6549617; doi:10.1136/bmjopen-2018-026554)
Supplement: Supplementary file 1 [file bmjopen-2018-026554supp001.pdf]

Supplementary file 1: PubMed search strategy

Financial risk protection: ("Financial risk protection" OR "Financial hardship " OR “Financial protection” OR “Financial protection in health” OR "Catastrophic health expenditure" OR “Catastrophic medical expenditure” OR "Catastrophic health expenditure risk" OR “Catastrophic medical expenditure risk” OR “Catastrophic health payment” OR “Catastrophic medical payment” OR “CHE” OR “CMP” OR “Medical induced poverty” OR “Health induced poverty” OR “Payment-induced poverty” OR “Catastrophic payment” OR "Catastrophic cost" OR "Health Impoverishment" OR "Medical Impoverishment" OR "Extended cost-effectiveness analysis" OR "ECEA")

AND

Methods used and their application: (“Universal health coverage” OR “UHC” OR “Health benefits package” OR “HBP” OR “Basic package of health services” OR “BPHS” OR “Essential package of health services” OR “EPHS” OR “Priority setting” OR “Health policy” OR “Resource allocation” OR “Allocative efficiency” OR “Methodology” OR “Measurement” OR “Modelling” OR “Distributional analysis” OR “Tracking” OR “Monitoring” OR “Estimating” OR “Quantifying” OR “Threshold” OR “Health financing” OR “Health insurance” OR “Social health insurance” OR “National health insurance” OR “National health programs”)

AND

Additional Limits: Humans[Mesh]
