# Supplementary material for: Measuring financial risk protection in health benefits packages: scoping review protocol to inform allocative efficiency studies
Source: BMJ Open. 2019 May 28;9(5):e026554. doi: 10.1136/bmjopen-2018-026554 (PMC6549617; doi:10.1136/bmjopen-2018-026554)
Supplement: Author's manuscript [file bmjopen-2018-026554.draft_revisions.pdf]

BMJ Open

BMJ Open is committed to open peer review. As part of this commitment we make the peer review history of every article we publish publicly available.

When an article is published we post the peer reviewers' comments and the authors' responses online. We also post the versions of the paper that were used during peer review. These are the versions that the peer review comments apply to.

The versions of the paper that follow are the versions that were submitted during the peer review process. They are not the versions of record or the final published versions. They should not be cited or distributed as the published version of this manuscript.

BMJ Open is an open access journal and the full, final, typeset and author-corrected version of record of the manuscript is available on our site with no access controls, subscription charges or pay-per-view fees (http://bmjopen.bmj.com).

If you have any questions on BMJ Open's open peer review process please email info.bmjopen@bmj.com

BMJ Open

Measuring Financial Risk Protection in Health Benefits Packages: A Scoping Review Protocol to Inform Allocative Efficiency Studies

Journal:	*BMJ Open*
Manuscript ID	bmjopen-2018-026554
Article Type:	Protocol
Date Submitted by the Author:	08-Sep-2018
Complete List of Authors:	Abou Jaoude, Gerard; University College London Institute for Global Health,<br>Skordis-Worrall, Jolene; University College London, Institute for Global Health<br>Haghparast-Bidgoli, Hassan; University College London, Institute for Global Health
Keywords:	Universal Health Coverage, Financial Risk Protection, Priority Setting, Allocative Efficiency Modelling, Scoping Review Protocol

SCHOLARONE™
Manuscripts

For peer review only

Measuring Financial Risk Protection in Health Benefits Packages: A Scoping Review Protocol to Inform Allocative Efficiency Studies

Authors: Gerard Joseph Abou Jaoude[1], Jolene Skordis[1], Hassan Haghparast-Bidgoli[1]

GAJ – Research Associate
JS – Reader
HHB – Principal Research Fellow

Affiliations:

[1]Institute for Global Health, University College London (UCL), Institute for Child Health, 30 Guilford Street, London WC1N 1EH, United Kingdom.

Correspondence to: Gerard Joseph Abou Jaoude, gerard.jaoude.15@ucl.ac.uk, Institute for Child Health, 30 Guilford Street, London WC1N 1EH, +44 (0)20 7905 2779.

Word count (excluding title page, abstract, references, figures and tables): 2414

For peer review only

ABSTRACT

Introduction: To progress toward Universal Health Coverage (UHC), countries will need to define a health benefits package of services free at the point of use. Financial risk protection is a core component of UHC, and should therefore be considered a key dimension of health benefits packages. Allocative efficiency modelling tools can support national analytical capacity to inform an evidence-based selection of services, but none are currently able to estimate financial risk protection. A review of existing methods used to measure financial risk protection can facilitate their inclusion in modelling tools, so that the latter can become more relevant to national decision making in light of UHC.

Methods and analysis: This protocol proposes to conduct a scoping review of existing methods used to measure financial risk protection, and assess their potential to inform the selection of services in a health benefits package. The proposed review will follow the methodological framework developed by Arksey and O'Malley and the subsequent recommendations made by Levac et al. Several databases will be systematically searched including: (1) PubMed; (2) Scopus; (3) Web of Science; and (4) Google Scholar. Grey literature will also be scanned and the bibliography of all selected studies will be hand searched. Following the selection of studies according to defined inclusion and exclusion criteria, key characteristics will be collected from the studies using a data extraction tool. Key characteristics will include the type of method used, geographical region of focus, and application to specific services or packages. The extracted data will then be charted, collated, reported and summarised using descriptive statistics, a thematic analysis, and graphical presentations.

Ethics and dissemination: The scoping review proposed in this protocol does not require ethical approval. The final results will be disseminated via publication in a peer-reviewed journal, conference presentations, and shared with key stakeholders.

ARTICLE SUMMARY

Strengths and limitations of this study

- This protocol proposes to conduct the first scoping review of methods used to measure financial risk protection
- A systematic search strategy will be applied to four key electronic databases and grey literature will be scanned
- The proposed review aims to not only outline financial risk protection measurement methods, but also detail their application to specific services or packages to assess how they could be incuded in allocative efficiency modelling
- A consultation stage is proposed in this protocol, which can improve the comprehensiveness and relevance of the final review

INTRODUCTION

For peer review only

Background

The political and academic focus on Universal Health Coverage (UHC) continues to grow globally, as highlighted by the inclusion of UHC as target 3.8 in the UN Sustainable Development Goals (SDGs).[1] According to the World Health Organisation (WHO), "UHC means that all individuals and communities receive the health services they need without suffering financial hardship".[2] This definition by the WHO is not prescriptive, but is clear in outlining that UHC does not involve providing all possible health services free of charge for everyone. Instead, UHC entails the provision of a defined package of effective services that are of sufficient quality and maximise health, equity and protection from financial hardship.[3-5]

Given the resource constraints that countries face, particularly low- and middle-income countries, challenging decisions will need to be made on what services will be provided free of charge and which services will not.[6-7] When working toward UHC, governments will therefore have to define a health benefits package that is free at the point of use, sometimes referred to as a basic or essential package of health services. Health financing reforms, and how best to implement selected services to maximise quality and access, will also need to be considered.[6-10] Best practice in priority setting commonly involves a transparent, systematic and evidence-based approach with high levels of stakeholder involvement.[6,11] Due to financial and human resource constraints, countries seeking to embark on these complex health system reforms will require administrative and analytic support for evidence-based decision making.[6,12-13]

Modelling tools can help support national analytical capacity and facilitate evidence-based priority setting.[6,14-15] Allocative efficiency analyses may be especially useful in this context as they are able to estimate and compare the health impact of various packages of services, with different budgets, while considering trade-offs between desired objectives – for example, minimising disease incidence or mortality.[16-17]

To date, allocative efficiency analyses have commonly been used to inform decision making for national disease control programmes, such as National TB or HIV Programmes,[15,18-19] with some efforts to estimate the distributional impacts of different responses.[20] There is largely unexplored potential for allocative efficiency analyses to inform priority-setting across diseases, while considering the costs, effectiveness, equity impact and financial risk protection of health benefits packages. The literature on how to consider costs and effectiveness is well established, but there is less research on the inclusion of equity measures,[21-22] and to our knowledge, financial risk protection has not yet been incorporated into allocative efficiency modelling analyses.

Rationale

It is estimated that 808 million people incurred catastrophic health expenditures in 2010.[23] Financial risk protection to avert catastrophic health expenditure and medical impoverishment is a core component of UHC. It is explicitly referred to in target 3.8 of the SDGs, but countries are not on track to meet targets for financial risk protection.[24] When defining or updating national health benefits packages, governments should therefore

For peer review only

consider financial risk protection as a key dimension of the priority setting process. If allocative efficiency and modelling analyses are to inform policies for UHC and the contents of health benefits packages, a method to estimate financial risk protection must be incorporated into the analyses. A scoping review of financial risk protection measurement methods, and how they could be applied to allocative efficiency analyses, can provide a foundation for modelling efforts to become more relevant in light of the ongoing push toward UHC. No such review has yet been conducted and this protocol proposes to address this gap.

Objectives

The objectives of the proposed scoping review are to outline the different methods used to measure financial risk protection, and discuss their application to specific services or health packages. Subsequently, the review will also seek to consider how existing financial risk protection measurement methods could be applied to allocative efficiency analyses of:

1. Disease-specific health packages
2. Health benefits packages that address multiple diseases

METHODS AND ANALYSIS

Scoping reviews benefit from a breadth of content by providing an overview of all the research in a given subject area. They also achieve depth through the mapping and interpretation of research.[25] The objectives of the proposed review lend themselves well to such an approach. They are broad in their scope, but precise in their aim to map existing research on financial risk protection measurement methods while considering how they could be applied to allocative efficiency analyses. The proposed scoping review will follow the six stages outlined in the framework by Arksey and O'Malley,[25] hereinafter referred to as Arksey and O'Malley's framework, which are discussed in detail below. The recommendations by Levac et al.,[26] who further clarify and build on Arksey and O'Malley's framework, will also be incorporated throughout the review process.

Stage 1: Identifying the research questions

The overarching question of the proposed review will guide the search strategy and the interpretation and reporting of the results: What are the different methods currently used to measure financial risk protection? This question should be broad enough to ensure that all the existing literature is captured and analysed in the review. Two sub-questions have also been identified, which will focus on the application of existing methods to health services or packages, and their potential to be included in allocative efficiency analyses. The two sub-questions are as follows: (1) How have different methods been used to estimate financial risk protection for health services or packages? (2) How could existing financial risk protection measurement methods be applied to allocative efficiency analyses? These questions will be subjected to iterative thinking as the review is carried out. While the overarching question is unlikely to change, the sub-questions proposed may be refined or additional questions included.

For peer review only

Stage 2: Identifying relevant studies

Stage 2 of the scoping review will involve identifying relevant studies for selection. A systematic search strategy, which is guided by the overarching question and its sub-components, will be used to carry out this stage of the review. The following electronic databases will be searched: (1) PubMed; (2) Scopus; (3) Web of Science; and (4) Google Scholar. The initial set of search terms and strategy proposed in table 1 and supplementary file 1 respectively, generates 966 results on PubMed. The search terms and search strategy have been formulated to identify research on the measurement of financial risk protection, and how the different methods have been applied. Search results will be downloaded and imported using EndNote, and duplicates will be deleted prior to screening according to the inclusion and exclusion criteria set in Stage 3.

Table 1: List of search terms and search strategy

Search terms for financial risk protection	Search terms for methods used and their application
"Financial risk protection" OR<br>"Financial hardship " OR<br>“Financial protection” OR<br>"Catastrophic health expenditure" OR<br>“Catastrophic medical expenditure” OR<br>"Catastrophic health expenditure risk" OR<br>“Catastrophic medical expenditure risk” OR<br>“Catastrophic health payment” OR<br>“Catastrophic payment” OR<br>"Health Impoverishment" OR<br>"Medical Impoverishment" OR<br>"Extended cost-effectiveness analysis" OR<br>"ECEA"	“Universal health coverage” OR<br>“UHC” OR<br>“Health benefits package” OR<br>“HBP” OR<br>“Basic package of health services” OR<br>“BPHS” OR<br>“Essential package of health services” OR<br>“EPHS” OR<br>“Priority setting” OR<br>“Health policy” OR<br>“Resource allocation” OR<br>“Allocative efficiency” OR<br>“Methodology” OR<br>“Measurement” OR<br>“Modelling” OR<br>“Distributional analysis” OR<br>“Tracking” OR<br>“Monitoring” OR<br>“Estimating” OR<br>“Quantifying” OR<br>“Threshold” OR<br>“Healthcare financing” OR<br>“Health insurance” OR<br>“Social health insurance” OR<br>“National health insurance”<br>“National health Programs”

Grey literature from institutions or organisations working on financial risk protection and UHC will also be scanned. Examples of such institutions or organisations are the World Health Organisation (WHO), World Bank, Disease Control Priorities Network, Centre for Global Development, International Decision Support initiative (iDSI), Health Policy Plus

For peer review only - http://bmjopen.bmj.com/site/about/guidelines.xhtml

(HP+), United Nations Department of Economic and Social Affairs (UN DESA), and the Health Intervention and Technology Assessment Program (HITAP). The reference lists of all literature deemed relevant according to the inclusion and exclusion criteria from Stage 3 will then be hand-searched. In line with Arksey and O'Malley's framework and Levac et al., the search terms and search strategy will be improved in an iterative process as we become more familiar with the literature.

Stage 3: Selecting studies

The third stage of the review will involve setting inclusion and exclusion criteria. Preliminary inclusion and exclusion criteria are proposed below, but these will be subjected to iterative thinking as specified by Arksey and O'Malley. Studies generated by the search will then be screened according to the inclusion and exclusion criteria in a two-step selection process. The first screen will be based on the relevance of the titles and abstracts, followed by a second screen after reading the remaining articles in full.

Inclusion criteria

Peer-reviewed journal articles and grey literature measuring or discussing methodologies for measuring financial risk protection will be included. Research estimating or conceptualising financial risk protection using either quantitative or qualitative techniques, regardless of the year published, geographic location, disease area of focus, health services or population groups considered will also be included.

Exclusion criteria

Papers that lack discussion either on the methodology behind the measures, their application to health services, or implications for priority setting will be excluded. Papers that cannot be accessed through institutional log-in, or in languages other than English and French will also be excluded.

Stage 4: Charting the data

The data from studies identified and selected for final inclusion in Stage 3, will be collected and charted. A data collection tool will be developed to collect key characteristics from the journal papers and grey literature included, such as the methods used, application to health services or packages, and country or region of interest. The tool will be developed in an iterative manner during the data collection process. Any decisions to amend the tool during the data collection process, and any changes to the overall review protocol will be documented and reported appropriately. Both descriptive statistics and qualitative analyses will then be carried out on the data collected before reporting the results.

Data will be aggregated and charted according to the different characteristic collected. For example, the number of studies conducted by or concerned with a given type of method, or by geographic region of interest. While there is an ongoing initiative to track progress toward UHC and financial risk protection on a global scale,[27-30] this does not map the amount of research conducted by geographic region. Therefore, using the data extracted we

For peer review only

will attempt to compare the amount of research conducted on financial risk protection with the burden of financial hardship by geographic region. Given that the focus of the proposed review is on mapping the different methodologies available to measure financial risk protection, a thematic analysis will also be undertaken to summarise and better understand the different approaches and any overlap or links between them.

Stage 5: Collating, summarising and reporting the results

The data collected in Stage 4 will be collated, summarised and reported in order to effectively map the different methods currently used to measure financial risk protection. Papers on the different methods used to measure financial risk protection will be classified according to the different characteristics identified and gathered through data extraction and charting. Tables and graphical presentations such as bar charts will be used to present the data in an aggregated manner, to provide an overview of the methods and how they could be included in allocative efficiency modelling. The latter will be assessed according to certain charactericts collected for different measurement methods, such as data requirements and application to individual health services or packages.

Stage 6: Consultation

The consultation stage aims to involve specialists in financial risk protection and allocative efficiency modelling, as these will likely be the primary target audience of the work. The consultation stage was described as optional but desirable by Arksey and O'Malley. Since then, the recommendations outlined by Levac et al. have suggested this should be a required step as it adds methodological rigour. In line with the recommendation to establish a clear purpose for the consultation, the following two objectives are proposed due to their potential to strengthen the review. First, to ascertain whether any methods or approaches to measure financial risk protection have not been captured by the review. Second, to assess the accuracy and instructiveness of the review and the results reported.

Study timeline

The first and second stages of this study started in June 2018 and were required to develop this protocol. Stage 3 of the review will commence in October 2018 and will take five months to complete by February 2019. The fourth stage is expected to take two months and finish in April 2019. An additional two months have been assigned for Stages 5 and 6, and for reporting the results. The estimated completion date of this scoping review is therefore June 2019.

Patient and public involvement

Patients and the public were not involved in the development of this protocol. If specialists agree to be involved in the consultation stage, details of this will be included in the final scoping review.

DISCUSSION (ETHICS AND DISSEMINATION)

For peer review only

Upon completion of all the steps outlined in this protocol, the findings of the proposed scoping review will be disseminated through the submission of a paper for peer-reviewed publication and conference presentations. The findings will also be shared with the World Health Organisation, the Disease Control Priorities Network, and other organisations working to implement and estimate the impact of health benefits packages in a move toward UHC or that are involved in allocative efficiency modelling. This study does not require ethical approval as only secondary data will be used.

The global movement toward UHC is accelerating, and as governments undertake policy reforms it is essential that the decisions made are transparent, systematic and evidence-based. Allocative efficiency analyses can inform such reforms through a systematic and evidence-based approach. However, measures of financial risk protection will need to be incorporated into allocative efficiency modelling if the efforts are to be useful and relevant to decision makers. The proposed scoping review outlined in this protocol can enable the latter by mapping the different available approaches for measuring financial risk protection, and discussing their suitability for analyses seeking to inform priority setting in health.

AUTHOR CONTRIBUTIONS

All authors contributed meaningfully to the preparation, drafting and editing of this scoping review protocol. GAJ and HHB conceived the idea and GAJ led protocol development. GAJ (corresponding author) conceptualised the research questions and core research plan details, before preparing the initial draft of this manuscript. JS and HHB provided critical input on the draft, research questions, and methodology. All authors approved the final submitted manuscript, after several iterations and rounds of editing, and agree to be accountable for all aspects of this protocol.

ACKNOWLEDGEMENTS

Not applicable.

DATA SHARING STATEMENT

There are no additional data available.

FUNDING

This work was supported by the Bill and Melinda Gates Foundation grant number OPP1179683.

COMPETING INTERESTS

For peer review only

All authors declare they have no competing interests.

PATIENT CONSENT

None required.

REFERENCES

1. United Nations. https://sustainabledevelopment.un.org/ (accessed 19 August 2018).
2. World Health Organisation. Universal Health Coverage: Fact Sheet. *World Health Organisation* 2017 http://www.who.int/news-room/fact-sheets/detail/universal-health-coverage-(uhc) (accessed 19 August 2018).
3. World Health Organisation. The world health report: health systems financing: the path to universal coverage. *Geneva: World Health Organisation* 2010.
4. World Health Organisation. Making fair choices on the path to universal health coverage. *Geneva: World Health Organisation* 2014.
5. Norheim OF. Ethical priority setting for universal health coverage: challenges in deciding upon fair distribution of health services. *BMC Med* 2016 Dec;14:75.
6. Glassman A, Giedion U, Smith PC, eds. What's in, what's out: designing benefits for universal health coverage. *Washington, D.C: Center for Global Development,* 2017
7. Jamison DT, Gelband H, Horton S, *et al.*, eds. Disease Control Priorities, (Volume 9): Improving Health and Reducing Poverty. *The World Bank* 2017
8. Peters DH, Garg A, Bloom G, *et al*. Poverty and access to health care in developing countries. *Ann N Y Acad Sci* 2008;1136:161-71.
9. Dieleman JL, Sadat N, Chang AY, *et al*. Trends in future health financing and coverage: future health spending and universal health coverage in 188 countries, 2016–40. *Lancet* 2018;391:1783-98.
10. Chalkidou K, Glassman A, Marten R, *et al*. Priority-setting for achieving universal health coverage. *Bull World Health Organ* 2016;94:462.
11. Barasa EW, Molyneux S, English M, *et al*. Setting healthcare priorities at the macro and meso levels: a framework for evaluation. *Int J Health Policy Manag* 2015;4:719.
12. Nuyens Y. Setting priorities for health research: lessons from low- and middle-income countries. *Bull World Health Organ* 2007;85:319-321.
13. Glassman A, Giedion U, Sakuma Y, *et al*. Defining a health benefits package: what are the necessary processes?. *Health Syst Reform* 2016;2:39-50.
14. Baltussen R, Niessen L. Priority setting of health interventions: the need for multi-criteria decision analysis. *Cost Eff Resour Alloc* 2006;4:14.
15. Stuart RM, Grobicki L, Haghparast-Bidgoli H, *et al.* How should HIV resources be allocated? Lessons learnt from applying Optima HIV in 23 countries. *J Int AIDS Soc* 2018;21:e25097.
16. Kerr CC, Stuart RM, Gray RT, *et al*. Optima: a model for HIV epidemic analysis, program prioritization, and resource optimization. *J Acquir Immune Defic Syndr* 2015;69:365-76.

17. Pearson R, Killedar M, Petravic J, *et al.* Optima Nutrition: an allocative efficiency tool to reduce childhood stunting by better targeting of nutrition-related interventions. *BMC Public Health* 2018;18:384.
18. Kelly SL, Martin-Hughes R, Stuart RM, *et al*. The global Optima HIV allocative efficiency model: targeting resources in efforts to end AIDS. *Lancet HIV* 2018;5:e190-8.
19. Stuart RM, Kerr CC, Haghparast-Bidgoli H, *et al*. Getting it right when budgets are tight: Using optimal expansion pathways to prioritize responses to concentrated and mixed HIV epidemics. *PLoS One*. 2017;12:e0185077.
20. Cleary S, Mooney G, McIntyre D. Equity and efficiency in HIV-treatment in South Africa: the contribution of mathematical programming to priority setting. *Health Econ* 2010;19:1166-80.
21. Norheim OF, Baltussen R, Johri M, *et al*. Guidance on priority setting in health care (GPS-Health): the inclusion of equity criteria not captured by cost-effectiveness analysis. *Cost Eff Resour Alloc* 2014;12:18.
22. Baltussen R, Jansen MP, Mikkelsen E, *et al*. Priority setting for universal health coverage: we need evidence-informed deliberative processes, not just more evidence on cost-effectiveness. *Int J Health Policy Manag* 2016;5:615.
23. Wagstaff A, Flores G, Hsu J, *et al*. Progress on catastrophic health spending in 133 countries: a retrospective observational study. *Lancet Glob Health* 2018;6:e169-79.
24. Verguet S, Woldemariam AT, Durrett WN, *et al*. Is the sustainable development goal target for financial risk protection in health realistic?. *BMJ Glob Health* 2017;2:e000216.
25. Arksey H, O'Malley L. Scoping studies: towards a methodological framework. *Int J Soc Res Methodol* 2005;8:19-32.
26. Levac D, Colquhoun H, O'Brien KK. Scoping studies: advancing the methodology. *Implement Sci* 2010;5:69.
27. World Health Organisation. Global Health Observatory. http://apps.who.int/gho/portal/uhc-fp-cabinet-wrapper-v2.jsp?id=1020201 (accessed 19 August 2018). (accessed 19 August 2018).
28. Boerma T, Eozenou P, Evans D, *et al*. Monitoring progress towards universal health coverage at country and global levels. *PLoS Med* 2014;11:e1001731.
29. World Health Organization, World Bank Group. Monitoring progress towards universal health coverage at country and global levels: framework, measures and targets. *Geneva, Washington DC: World Health Organization, World Bank Group* 2014
30. World Health Organization, World Bank Group. Tracking universal health coverage: 2017 global monitoring report. *Geneva, Washington DC: World Health Organization, World Bank Group* 2017

For peer review only

Supplementary file 1: PubMed search strategy

Financial risk protection: ("Financial risk protection" OR "Financial hardship " OR “Financial protection” OR "Catastrophic health expenditure" OR “Catastrophic medical expenditure” OR "Catastrophic health expenditure risk" OR “Catastrophic medical expenditure risk” OR “Catastrophic health payment” OR “Catastrophic payment” OR "Health Impoverishment" OR "Medical Impoverishment" OR "Extended cost-effectiveness analysis" OR "ECEA")

AND

Methods used and their application: (“Universal health coverage” OR “UHC” OR “Health benefits package” OR “HBP” OR “Basic package of health services” OR “BPHS” OR “Essential package of health services” OR “EPHS” OR “Priority setting” OR “Health policy” OR “Resource allocation” OR “Allocative efficiency” OR “Methodology” OR “Measurement” OR “Modelling” OR “Distributional analysis” OR “Tracking” OR “Monitoring” OR “Estimating” OR “Quantifying” OR “Threshold” OR “Health financing” OR “Health insurance” OR “Social health insurance” OR “National health insurance” OR “National health programs”)

AND

Additional Limits: Humans[Mesh]

For peer review only

BMJ Open

Measuring Financial Risk Protection in Health Benefits Packages: A Scoping Review Protocol to Inform Allocative Efficiency Studies

Journal:	*BMJ Open*
Manuscript ID	bmjopen-2018-026554.R1
Article Type:	Protocol
Date Submitted by the Author:	29-Mar-2019
Complete List of Authors:	Abou Jaoude, Gerard; University College London Institute for Global Health,<br>Skordis-Worrall, Jolene; University College London, Institute for Global Health<br>Haghparast-Bidgoli, Hassan; University College London, Institute for Global Health
**Primary Subject Heading**:	Health economics
Secondary Subject Heading:	Global health, Health policy
Keywords:	Universal Health Coverage, Financial Risk Protection, Priority Setting, Allocative Efficiency Modelling, Scoping Review Protocol

SCHOLARONE™
Manuscripts

Measuring Financial Risk Protection in Health Benefits Packages: A Scoping Review Protocol to Inform Allocative Efficiency Studies

Authors: Gerard Joseph Abou Jaoude[1], Jolene Skordis[1], Hassan Haghparast-Bidgoli[1]

GAJ – Research Associate
JS – Reader
HHB – Principal Research Fellow

Affiliations:

[1]Institute for Global Health, University College London (UCL), Institute for Child Health, 30 Guilford Street, London WC1N 1EH, United Kingdom.

Correspondence to: Gerard Joseph Abou Jaoude, gerard.jaoude.15@ucl.ac.uk, Institute for Child Health, 30 Guilford Street, London WC1N 1EH, +44 (0)20 7905 2779.

Word count (excluding title page, abstract, references, figures and tables): 2,637

For peer review only

ABSTRACT

Introduction: To progress toward Universal Health Coverage (UHC), countries will need to define a health benefits package of services free at the point of use. Financial risk protection is a core component of UHC, and should therefore be considered a key dimension of health benefits packages. Allocative efficiency modelling tools can support national analytical capacity to inform an evidence-based selection of services, but none are currently able to estimate financial risk protection. A review of existing methods used to measure financial risk protection can facilitate their inclusion in modelling tools, so that the latter can become more relevant to national decision making in light of UHC.

Methods and analysis: This protocol proposes to conduct a scoping review of existing methods used to measure financial risk protection, and assess their potential to inform the selection of services in a health benefits package. The proposed review will follow the methodological framework developed by Arksey and O'Malley and the subsequent recommendations made by Levac et al. Several databases will be systematically searched including: (1) PubMed; (2) Scopus; (3) Web of Science; and (4) Google Scholar. Grey literature will also be scanned and the bibliography of all selected studies will be hand searched. Following the selection of studies according to defined inclusion and exclusion criteria, key characteristics will be collected from the studies using a data extraction tool. Key characteristics will include the type of method used, geographical region of focus, and application to specific services or packages. The extracted data will then be charted, collated, reported and summarised using descriptive statistics, a thematic analysis, and graphical presentations.

Ethics and dissemination: The scoping review proposed in this protocol does not require ethical approval. The final results will be disseminated via publication in a peer-reviewed journal, conference presentations, and shared with key stakeholders.

ARTICLE SUMMARY

Strengths and limitations of this study

- This protocol proposes to conduct the first scoping review of methods used to measure financial risk protection
- A systematic search strategy will be applied to four key electronic databases and grey literature will be scanned
- The proposed review aims to not only outline financial risk protection measurement methods, but also detail their application to specific services or packages to assess how they could be included in allocative efficiency modelling
- A consultation stage is proposed in this protocol, which can improve the comprehensiveness and relevance of the final review

INTRODUCTION

For peer review only - http://bmjopen.bmj.com/site/about/guidelines.xhtml

Background

The political and academic focus on Universal Health Coverage (UHC) continues to grow globally, as highlighted by the inclusion of UHC as target 3.8 in the UN Sustainable Development Goals (SDGs).[1] According to the World Health Organisation (WHO), "UHC means that all individuals and communities receive the health services they need without suffering financial hardship".[2] This definition by the WHO is not prescriptive, but is clear in outlining that UHC does not involve providing all possible health services free of charge for everyone. Instead, UHC entails the provision of a defined package of effective services that are of sufficient quality and maximise health, equity and protection from financial hardship.[3-5]

Given the resource constraints that countries face, particularly low- and middle-income countries, challenging decisions will need to be made on what services will be provided free of charge and which services will not.[6-7] When working toward UHC, governments will therefore have to define a health benefits package that is free at the point of use, sometimes referred to as a basic or essential package of health services. Health financing reforms, and how best to implement selected services to maximise quality and access, will also need to be considered.[6-10] Best practice in priority setting commonly involves a transparent, systematic and evidence-based approach with high levels of stakeholder involvement.[6,11] Due to financial and human resource constraints, countries seeking to embark on these complex health system reforms will require administrative and analytic support for evidence-based decision making.[6,12-13]

Modelling tools can help support national analytical capacity and facilitate evidence-based priority setting.[6,14-15] Allocative efficiency analyses may be especially useful in this context as they are able to estimate and compare the health impact of various packages of services, with different budgets, while considering trade-offs between desired objectives – for example, minimising disease incidence or mortality.[16-17]

To date, allocative efficiency analyses have commonly been used to inform decision making for national disease control programmes, such as National TB or HIV Programmes.[15,18-19] There is largely unexplored potential for allocative efficiency analyses to inform priority-setting across diseases, while considering the costs, effectiveness, equity impact and financial risk protection of health benefits packages. The literature on how to consider costs and effectiveness is well established, but there is less research on the inclusion of equity measures,[20-22] and to our knowledge, financial risk protection has not yet been incorporated into allocative efficiency modelling analyses.

Rationale

Links between poverty and health have been studied extensively, highlighting the importance of socio-economic status and income inequality as determinants of health.[23-25] Likewise, indebtedness due to medical expenses is one of the main pathways into and a major cause of remaining trapped in poverty.[26-28] For individuals or households, particularly those with low income, medical expenses due to health shocks can put at risk or negatively impact non-medical consumption causing financial strain or hardship.[29-30]

For peer review only - http://bmjopen.bmj.com/site/about/guidelines.xhtml

Financial risk protection can therefore be understood as protection against the latter by the partial or full subsidisation of healthcare costs through mechanisms such as public financing and formal, informal or self-provided insurance.[31] Several measures of financial risk protection currently exist but the most frequently used indicators, adopted both by the WHO and World Bank, are measures of financial hardship and can be categorised into catastrophic health expenditure and impoverishment. Catastrophic health expenditure occurs when the proportion of a household's medical expenses relative to its income or capacity to pay exceeds a defined threshold.[32-34] Measures of impoverishment on the other hand, aim to capture the number of households pushed into, or deeper into, poverty due to healthcare costs.[34-35]

It is estimated that 808 million people incurred catastrophic health expenditures in 2010.[36] Financial risk protection to avert catastrophic health expenditure and medical impoverishment is a core component of UHC. It is explicitly referred to in target 3.8 of the SDGs, but countries are not on track to meet targets for financial risk protection.[37] When defining or updating national health benefits packages, governments should therefore consider financial risk protection as a key dimension of the priority setting process alongside the maximisation of health benefits. If allocative efficiency and modelling analyses are to inform policies for UHC and the contents of health benefits packages, a method to estimate financial risk protection must be incorporated into the analyses. A scoping review of financial risk protection measurement methods, and how they could be applied to allocative efficiency analyses, can provide a foundation for modelling efforts to become more relevant in light of the ongoing push toward UHC. No such review has yet been conducted and this protocol proposes to address this gap.

Objectives

The primary objective of the proposed scoping review is to outline the different methods used to measure financial risk protection, and discuss their strengths and weaknesses as well as application to specific services or health packages. Subsequently, the review will seek to consider how existing financial risk protection measurement methods could be applied to allocative efficiency analyses of:

1. Disease-specific health packages
2. Health benefits packages that address multiple diseases

The secondary objective of the review will be to build on the work carried out by the World Bank and WHO to track progress toward UHC and financial risk protection.[38-41] The review will compare the amount of research on financial risk protection by country and region against the burden of financial hardship to establish any mismatch between research focus and burden.

METHODS AND ANALYSIS

Scoping reviews benefit from a breadth of content by providing an overview of all the research in a given subject area. They also achieve depth through the mapping and interpretation of research.[42] The objectives of the proposed review lend themselves well to such an approach. They are broad in their scope, but precise in their aim to map existing

For peer review only - http://bmjopen.bmj.com/site/about/guidelines.xhtml

research on financial risk protection measurement methods while considering how they could be applied to allocative efficiency analyses. The proposed scoping review will follow the six stages outlined in the framework by Arksey and O'Malley,[42] hereinafter referred to as Arksey and O'Malley's framework, which are discussed in detail below. The recommendations by Levac et al.,[43] who further clarify and build on Arksey and O'Malley's framework, will also be incorporated throughout the review process. The review will comply with the PRISMA checklist recently developed for scoping reviews.[44]

Stage 1: Identifying the research questions

The overarching question of the proposed review will guide the search strategy and the interpretation and reporting of the results: What are the different methods currently used to measure financial risk protection? This question should be broad enough to ensure that all the existing literature is captured and analysed in the review. Two sub-questions have also been identified, which will focus on the application of existing methods to health services or packages, and their potential to be included in allocative efficiency analyses. The two sub-questions are as follows: (1) How have different methods been used to estimate financial risk protection for health services or packages? (2) How could existing financial risk protection measurement methods be applied to allocative efficiency analyses? These questions will be subjected to iterative thinking as the review is carried out. While the overarching question is unlikely to change, the sub-questions proposed may be refined or additional questions included.

Stage 2: Identifying relevant studies

Stage 2 of the scoping review will involve identifying relevant studies for selection. A systematic search strategy, which is guided by the overarching question and its sub-components, will be used to carry out this stage of the review. The following electronic databases will be searched: (1) PubMed; (2) Scopus; (3) Web of Science; and (4) Google Scholar. The initial set of search terms and strategy proposed in table 1 and supplementary file 1 respectively, generates 1594 results on PubMed. The search terms and search strategy have been formulated to identify research on the measurement of financial risk protection, and how the different methods have been applied. Search results will be downloaded and imported using EndNote, and duplicates will be deleted prior to screening according to the inclusion and exclusion criteria set in Stage 3.

Table 1: List of search terms and search strategy

Search terms for financial risk protection	Search terms for methods used and their application
"Financial risk protection" OR<br>"Financial hardship " OR<br>"Financial protection" OR<br>"Financial protection in health" OR<br>"Catastrophic health expenditure" OR<br>"Catastrophic medical expenditure" OR<br>"Catastrophic health expenditure risk" OR<br>"Catastrophic medical expenditure risk" OR	"Universal health coverage" OR<br>"UHC" OR<br>"Health benefits package" OR<br>"HBP" OR<br>"Basic package of health services" OR<br>"BPHS" OR<br>"Essential package of health services" OR<br>"EPHS" OR

For peer review only - http://bmjopen.bmj.com/site/about/guidelines.xhtml

“Catastrophic health payment” OR<br>“Catastrophic medical payment” OR<br>“CHE” OR<br>“CMP” OR<br>“Medical induced poverty” OR<br>“Health induced poverty” OR<br>“Payment-induced poverty” OR<br>“Catastrophic payment” OR<br>“Catastrophic cost” OR<br>"Health Impoverishment" OR<br>"Medical Impoverishment" OR<br>"Extended cost-effectiveness analysis" OR<br>"ECEA"	“Priority setting” OR<br>“Health policy” OR<br>“Resource allocation” OR<br>“Allocative efficiency” OR<br>“Methodology” OR<br>“Measurement” OR<br>“Modelling” OR<br>“Distributional analysis” OR<br>“Tracking” OR<br>“Monitoring” OR<br>“Estimating” OR<br>“Quantifying” OR<br>“Threshold” OR<br>“Healthcare financing” OR<br>“Health insurance” OR<br>“Social health insurance” OR<br>“National health insurance”<br>“National health programs”

Relevant grey literature on financial risk protection and UHC published by institutions or organisations working on these topics will also be scanned. Examples of such institutions or organisations are the World Health Organisation (WHO), World Bank, Disease Control Priorities Network, Centre for Global Development, International Decision Support initiative (iDSI), Health Policy Plus (HP+), United Nations Department of Economic and Social Affairs (UN DESA), and the Health Intervention and Technology Assessment Program (HITAP). The reference lists of all literature deemed relevant according to the inclusion and exclusion criteria from Stage 3 will then be hand-searched. In line with Arksey and O’Malley’s framework and Levac et al., the search terms and search strategy will be improved in an iterative process as we become more familiar with the literature.

Stage 3: Selecting studies

The third stage of the review will involve setting inclusion and exclusion criteria. Preliminary inclusion and exclusion criteria are proposed below, but these will be subjected to iterative thinking as specified by Arksey and O’Malley. Studies generated by the search will then be screened according to the inclusion and exclusion criteria in a two-step selection process. The first screen will be based on the relevance of the titles and abstracts, followed by a second screen after reading the remaining articles in full.

Inclusion criteria

Peer-reviewed journal articles and grey literature measuring or discussing methodologies for measuring financial risk protection will be included. Research estimating or conceptualising financial risk protection using either quantitative or qualitative techniques, regardless of the year published, geographic location, disease area of focus, health services or population groups considered will also be included.

For peer review only - http://bmjopen.bmj.com/site/about/guidelines.xhtml

Exclusion criteria

Papers that lack discussion either on the methodology behind the measures, their application to health services, or implications for priority setting will be excluded. Papers that cannot be accessed through institutional log-in, or in languages other than English and French will also be excluded.

Stage 4: Charting the data

The data from studies identified and selected for final inclusion in Stage 3, will be collected and charted. A data collection tool will be developed to collect key characteristics from the journal papers and grey literature included, such as the methods used, application to health services or packages, and country or region of interest. The tool will be developed in an iterative manner during the data collection process. Any decisions to amend the tool during the data collection process, and any changes to the overall review protocol will be documented and reported appropriately. Both descriptive statistics and qualitative analyses will then be carried out on the data collected before reporting the results.

Data will be aggregated and charted according to the different characteristic collected. For example, the number of studies conducted by or concerned with a given type of method, or by geographic region of interest. While there is an ongoing initiative to track progress toward UHC and financial risk protection on a global scale,[38-41] this does not map the amount of research conducted by geographic region. Therefore, using the data extracted we will attempt to compare the amount of research conducted on financial risk protection with the burden of financial hardship by geographic region. Given that the focus of the proposed review is on mapping the different methodologies available to measure financial risk protection, a thematic analysis will also be undertaken to summarise and better understand the different approaches and any overlap or links between them. The strengths, weaknesses, and the applications of different methodologies will then be discussed and investigated in the review based on the data extracted.

Stage 5: Collating, summarising and reporting the results

The data collected in Stage 4 will be collated, summarised and reported in order to effectively map the different methods currently used to measure financial risk protection. Papers on the different methods used to measure financial risk protection will be classified according to the different characteristics identified and gathered through data extraction and charting. Tables and graphical presentations such as bar charts will be used to present the data in an aggregated manner, to provide an overview of the methods and how they could be included in allocative efficiency modelling. The latter will be assessed according to certain characteristics collected for different measurement methods, such as data requirements and application to individual health services or packages.

Stage 6: Consultation

The consultation stage aims to involve specialists in financial risk protection and allocative efficiency modelling, as these will likely be the primary target audience of the work.

For peer review only - http://bmjopen.bmj.com/site/about/guidelines.xhtml

The consultation stage was described as optional but desirable by Arksey and O'Malley. Since then, the recommendations outlined by Levac et al. have suggested this should be a required step as it adds methodological rigour. In line with the recommendation to establish a clear purpose for the consultation, the following two objectives are proposed due to their potential to strengthen the review. First, to ascertain whether any methods or approaches to measure financial risk protection have not been captured by the review. Second, to assess the accuracy and instructiveness of the review and the results reported.

Study timeline

The first and second stages of this study started in June 2018 and were required to develop this protocol. Stage 3 of the review commenced in October 2018 and took five months to complete by February 2019. The hand-searching of included articles and the fourth stage are expected to take four months and finish by June 2019. An additional two months have been assigned for Stages 5 and 6, and for reporting the results. The estimated completion date of this scoping review is therefore August 2019.

Patient and public involvement

Patients and the public were not involved in the development of this protocol. If specialists agree to be involved in the consultation stage, details of this will be included in the final scoping review.

DISCUSSION (ETHICS AND DISSEMINATION)

Upon completion of all the steps outlined in this protocol, the findings of the proposed scoping review will be disseminated through the submission of a paper for peer-reviewed publication and conference presentations. The findings will also be shared with the World Health Organisation, the Disease Control Priorities Network, and other organisations working to implement and estimate the impact of health benefits packages in a move toward UHC or that are involved in allocative efficiency modelling. This study does not require ethical approval as only secondary data will be used.

The proposed scoping review will be the first to comprehensively map the status-quo of research on the measurement of financial risk protection, both in terms of methodological developments and empirical work. The extraction of data from included literature is another advantage given that data extraction does not always feature in scoping reviews.[45] However, there are key limitations to the proposed review. First, the different stages have and will continue to be undertaken by the corresponding author only, under the supervision of co-authors. This introduces a greater margin for error and potential bias, particularly for stages 3 and 4.[46-47] Second, while the strengths, weaknesses, and applications of different methodologies will be discussed, the quality of included studies will not be considered as would commonly be the case in systematic reviews.[45] Third, the review will fail to capture some relevant empirical work published in languages other than English or French.

For peer review only - http://bmjopen.bmj.com/site/about/guidelines.xhtml

The global movement toward UHC is accelerating, and as governments undertake policy reforms it is essential that the decisions made are transparent, systematic and evidence-based. Allocative efficiency analyses can inform such reforms through a systematic and evidence-based approach. However, measures of financial risk protection will need to be incorporated into allocative efficiency modelling if the efforts are to be useful and relevant to decision makers. The proposed scoping review outlined in this protocol can enable the latter by mapping the different available approaches for measuring financial risk protection, and discussing their suitability for analyses seeking to inform priority setting in health.

AUTHOR CONTRIBUTIONS

All authors contributed meaningfully to the preparation, drafting and editing of this scoping review protocol. GAJ and HHB conceived the idea and GAJ led protocol development. GAJ (corresponding author) conceptualised the research questions and core research plan details, before preparing the initial draft of this manuscript. JS and HHB provided critical input on the draft, research questions, and methodology. All authors approved the final submitted manuscript, after several iterations and rounds of editing, and agree to be accountable for all aspects of this protocol.

ACKNOWLEDGEMENTS

Not applicable.

DATA SHARING STATEMENT

There are no additional data available.

FUNDING

This work was supported by the Bill and Melinda Gates Foundation grant number OPP1179683.

COMPETING INTERESTS

All authors declare they have no competing interests.

PATIENT CONSENT

None required.

For peer review only

For peer review only - http://bmjopen.bmj.com/site/about/guidelines.xhtml

REFERENCES

1. United Nations. https://sustainabledevelopment.un.org/ (accessed 19 August 2018).
2. World Health Organisation. Universal Health Coverage: Fact Sheet. *World Health Organisation* 2017 http://www.who.int/news-room/fact-sheets/detail/universal-health-coverage-(uhc) (accessed 19 August 2018).
3. World Health Organisation. The world health report: health systems financing: the path to universal coverage. *Geneva: World Health Organisation* 2010.
4. World Health Organisation. Making fair choices on the path to universal health coverage. *Geneva: World Health Organisation* 2014.
5. Norheim OF. Ethical priority setting for universal health coverage: challenges in deciding upon fair distribution of health services. *BMC Med* 2016 Dec;14:75.
6. Glassman A, Giedion U, Smith PC, eds. What's in, what's out: designing benefits for universal health coverage. *Washington, D.C: Center for Global Development,* 2017
7. Jamison DT, Gelband H, Horton S, *et al.*, eds. Disease Control Priorities, (Volume 9): Improving Health and Reducing Poverty. *The World Bank* 2017
8. Peters DH, Garg A, Bloom G, *et al*. Poverty and access to health care in developing countries. *Ann N Y Acad Sci* 2008;1136:161-71.
9. Dieleman JL, Sadat N, Chang AY, *et al*. Trends in future health financing and coverage: future health spending and universal health coverage in 188 countries, 2016–40. *Lancet* 2018;391:1783-98.
10. Chalkidou K, Glassman A, Marten R, *et al*. Priority-setting for achieving universal health coverage. *Bull World Health Organ* 2016;94:462.
11. Barasa EW, Molyneux S, English M, *et al*. Setting healthcare priorities at the macro and meso levels: a framework for evaluation. *Int J Health Policy Manag* 2015;4:719.
12. Nuyens Y. Setting priorities for health research: lessons from low- and middle-income countries. *Bull World Health Organ* 2007;85:319-321.
13. Glassman A, Giedion U, Sakuma Y, *et al*. Defining a health benefits package: what are the necessary processes?. *Health Syst Reform* 2016;2:39-50.
14. Baltussen R, Niessen L. Priority setting of health interventions: the need for multi-criteria decision analysis. *Cost Eff Resour Alloc* 2006;4:14.
15. Stuart RM, Grobicki L, Haghparast-Bidgoli H, *et al.* How should HIV resources be allocated? Lessons learnt from applying Optima HIV in 23 countries. *J Int AIDS Soc* 2018;21:e25097.
16. Kerr CC, Stuart RM, Gray RT, *et al*. Optima: a model for HIV epidemic analysis, program prioritization, and resource optimization. *J Acquir Immune Defic Syndr* 2015;69:365-76.
17. Pearson R, Killedar M, Petravic J, *et al.* Optima Nutrition: an allocative efficiency tool to reduce childhood stunting by better targeting of nutrition-related interventions. *BMC Public Health* 2018;18:384.
18. Kelly SL, Martin-Hughes R, Stuart RM, *et al*. The global Optima HIV allocative efficiency model: targeting resources in efforts to end AIDS. *Lancet HIV* 2018;5:e190-8.
19. Stuart RM, Kerr CC, Haghparast-Bidgoli H, *et al*. Getting it right when budgets are tight: Using optimal expansion pathways to prioritize responses to concentrated and mixed HIV epidemics. *PLoS One*. 2017;12:e0185077.

For peer review only - http://bmjopen.bmj.com/site/about/guidelines.xhtml

20. Cleary S, Mooney G, McIntyre D. Equity and efficiency in HIV-treatment in South Africa: the contribution of mathematical programming to priority setting. *Health Econ* 2010;19:1166-80.
21. Norheim OF, Baltussen R, Johri M, *et al*. Guidance on priority setting in health care (GPS-Health): the inclusion of equity criteria not captured by cost-effectiveness analysis. *Cost Eff Resour Alloc* 2014;12:18.
22. Baltussen R, Jansen MP, Mikkelsen E, *et al*. Priority setting for universal health coverage: we need evidence-informed deliberative processes, not just more evidence on cost-effectiveness. *Int J Health Policy Manag* 2016;5:615.
23. Adler NE, Ostrove JM. Socioeconomic status and health: what we know and what we don't. *Ann N Y Acad Sci* 1999;1;896(1):3-15.
24. Marmot M. Status syndrome: How your social standing directly affects your health. *A&C Black* 2005.
25. Pickett KE, Wilkinson RG. Income inequality and health: a causal review. *Soc Sci Med* 2015;1;128:316-26.
26. van Damme W. Out-of-pocket health expenditure and debt in poor households: evidence from Cambodia. *Trop Med Int Health* 2004;9(2):273–280.
27. Krishna A. Pathways out of and into poverty in 36 villages of Andhra Pradesh, India. *World Dev* 2006;34(2):271–288.
28. Sen B. Drivers of escape and descent: changing household fortunes in rural Bangladesh. *World Dev* 2003;1;31(3):513-34.
29. Russell S. Ability to pay for health care: concepts and evidence. *Health Policy and Plan* 1996;11:219–237
30. Wagstaff A. The economic consequences of health shocks: evidence from Vietnam. *J Health Econ* 2007;26(1):82-100.
31. O'Donnell O. Financial Protection Against Medical Expense. Tinbergen Institute Discussion Papers. *Tinbergen Institute* 2019;10.
32. Berki SE. A look at catastrophic medical expenses and the poor. *Health Aff* 1986;5(4):138-45.
33. Xu K, Evans DB, Kawabata K, Zeramdini R, Klavus J, Murray CJ. Household catastrophic health expenditure: a multicountry analysis. *Lancet* 2003;362(9378):111-7
34. Wagstaff A, Doorslaer EV. Catastrophe and impoverishment in paying for health care: with applications to Vietnam 1993–1998. *Health Econ* 2003;12(11):921-33.
35. Van Doorslaer E, O'Donnell O, Rannan-Eliya RP, Somanathan A, Adhikari SR, Garg CC, Harbianto D, Herrin AN, Huq MN, Ibragimova S, Karan A. Effect of payments for health care on poverty estimates in 11 countries in Asia: an analysis of household survey data. *Lancet* 2006 Oct 14;368(9544):1357-64.
36. Wagstaff A, Flores G, Hsu J, *et al*. Progress on catastrophic health spending in 133 countries: a retrospective observational study. *Lancet Glob Health* 2018;6:e169-79.
37. Verguet S, Woldemariam AT, Durrett WN, *et al*. Is the sustainable development goal target for financial risk protection in health realistic?. *BMJ Glob Health* 2017;2:e000216.
38. World Health Organisation. Global Health Observatory. http://apps.who.int/gho/portal/uhc-fp-cabinet-wrapper-v2.jsp?id=1020201 (accessed 19 August 2018). (accessed 19 August 2018).

For peer review only

39. Boerma T, Eozenou P, Evans D, *et al*. Monitoring progress towards universal health coverage at country and global levels. *PLoS Med* 2014;11:e1001731.
40. World Health Organization, World Bank Group. Monitoring progress towards universal health coverage at country and global levels: framework, measures and targets. *Geneva, Washington DC: World Health Organization, World Bank Group* 2014.
41. World Health Organization, World Bank Group. Tracking universal health coverage: 2017 global monitoring report. *Geneva, Washington DC: World Health Organization, World Bank Group* 2017.
42. Arksey H, O'Malley L. Scoping studies: towards a methodological framework. *Int J Soc Res Methodol* 2005;8:19-32.
43. Levac D, Colquhoun H, O'Brien KK. Scoping studies: advancing the methodology. *Implement Sci* 2010;5:69.
44. Tricco AC, Lillie E, Zarin W, O'Brien KK, Colquhoun H, Levac D, Moher D, Peters MD, Horsley T, Weeks L, Hempel S. PRISMA extension for scoping reviews (PRISMA-ScR): checklist and explanation. *Ann Intern Med* 2018;169(7):467-73.
45. Armstrong R, Hall BJ, Doyle J, Waters E. 'Scoping the scope'of a cochrane review. *J Public Health* 2011;33(1):147-50.
46. Edwards P, Clarke M, DiGuiseppi C, Pratap S, Roberts I, Wentz R. Identification of randomized controlled trials in systematic reviews: accuracy and reliability of screening records. *Stat Med* 2002;21(11):1635-40.
47. Buscemi N, Hartling L, Vandermeer B, Tjosvold L, Klassen TP. Single data extraction generated more errors than double data extraction in systematic reviews. *J Clin Epidemiol* 2006;59(7):697-703.

For peer review only

Supplementary file 1: PubMed search strategy

Financial risk protection: ("Financial risk protection" OR "Financial hardship " OR “Financial protection” OR “Financial protection in health” OR "Catastrophic health expenditure" OR “Catastrophic medical expenditure” OR "Catastrophic health expenditure risk" OR “Catastrophic medical expenditure risk” OR “Catastrophic health payment” OR “Catastrophic medical payment” OR “CHE” OR “CMP” OR “Medical induced poverty” OR “Health induced poverty” OR “Payment-induced poverty” OR “Catastrophic payment” OR "Catastrophic cost" OR "Health Impoverishment" OR "Medical Impoverishment" OR "Extended cost-effectiveness analysis" OR "ECEA")

AND

Methods used and their application: (“Universal health coverage” OR “UHC” OR “Health benefits package” OR “HBP” OR “Basic package of health services” OR “BPHS” OR “Essential package of health services” OR “EPHS” OR “Priority setting” OR “Health policy” OR “Resource allocation” OR “Allocative efficiency” OR “Methodology” OR “Measurement” OR “Modelling” OR “Distributional analysis” OR “Tracking” OR “Monitoring” OR “Estimating” OR “Quantifying” OR “Threshold” OR “Health financing” OR “Health insurance” OR “Social health insurance” OR “National health insurance” OR “National health programs”)

AND

Additional Limits: Humans[Mesh]

For peer review only

Measuring Financial Risk Protection in Health Benefits Packages: A Scoping Review Protocol to Inform Allocative Efficiency Studies

Journal:	*BMJ Open*
Manuscript ID	bmjopen-2018-026554.R2
Article Type:	Protocol
Date Submitted by the Author:	19-Apr-2019

Complete List of Authors:	Abou Jaoude, Gerard; University College London Institute for Global Health,<br>Skordis-Worrall, Jolene; University College London, Institute for Global Health<br>Haghparast-Bidgoli, Hassan; University College London, Institute for Global Health

**Primary Subject Heading**:	Health economics
Secondary Subject Heading:	Global health, Health policy
Keywords:	Universal Health Coverage, Financial Risk Protection, Priority Setting, Allocative Efficiency Modelling, Scoping Review Protocol

Measuring Financial Risk Protection in Health Benefits Packages: A Scoping Review Protocol to Inform Allocative Efficiency Studies

Authors: Gerard Joseph Abou Jaoude[1], Jolene Skordis[1], Hassan Haghparast-Bidgoli[1]

GAJ – Research Associate
JS – Reader
HHB – Principal Research Fellow

Affiliations:

[1]Institute for Global Health, University College London (UCL), Institute for Child Health, 30 Guilford Street, London WC1N 1EH, United Kingdom.

Correspondence to: Gerard Joseph Abou Jaoude, gerard.jaoude.15@ucl.ac.uk, Institute for Child Health, 30 Guilford Street, London WC1N 1EH, +44 (0)20 7905 2779.

Word count (excluding title page, abstract, references, figures and tables): 2,676

For peer review only

For peer review only - http://bmjopen.bmj.com/site/about/guidelines.xhtml

ABSTRACT

Introduction: To progress toward Universal Health Coverage (UHC), countries will need to define a health benefits package of services free at the point of use. Financial risk protection is a core component of UHC, and should therefore be considered a key dimension of health benefits packages. Allocative efficiency modelling tools can support national analytical capacity to inform an evidence-based selection of services, but none are currently able to estimate financial risk protection. A review of existing methods used to measure financial risk protection can facilitate their inclusion in modelling tools, so that the latter can become more relevant to national decision making in light of UHC.

Methods and analysis: This protocol proposes to conduct a scoping review of existing methods used to measure financial risk protection, and assess their potential to inform the selection of services in a health benefits package. The proposed review will follow the methodological framework developed by Arksey and O'Malley and the subsequent recommendations made by Levac et al. Several databases will be systematically searched including: (1) PubMed; (2) Scopus; (3) Web of Science; and (4) Google Scholar. Grey literature will also be scanned and the bibliography of all selected studies will be hand searched. Following the selection of studies according to defined inclusion and exclusion criteria, key characteristics will be collected from the studies using a data extraction tool. Key characteristics will include the type of method used, geographical region of focus, and application to specific services or packages. The extracted data will then be charted, collated, reported and summarised using descriptive statistics, a thematic analysis, and graphical presentations.

Ethics and dissemination: The scoping review proposed in this protocol does not require ethical approval. The final results will be disseminated via publication in a peer-reviewed journal, conference presentations, and shared with key stakeholders.

ARTICLE SUMMARY

Strengths and limitations of this study

- This protocol proposes to conduct the first scoping review of methods used to measure financial risk protection
- The proposed review aims to not only outline financial risk protection measurement methods, but also detail their application to specific services or packages to assess how they could be included in allocative efficiency modelling
- The review will be carried out by the corresponding author only, under the supervision of co-authors, which introduces a greater margin for error and potential bias for the screening and data extraction stages
- While the strengths, weaknesses, and applications of different methodologies will be discussed, the quality of included studies will not be considered
- A consultation stage is proposed in this protocol, which can improve the comprehensiveness and relevance of the final review

For peer review only - http://bmjopen.bmj.com/site/about/guidelines.xhtml

INTRODUCTION

Background

The political and academic focus on Universal Health Coverage (UHC) continues to grow globally, as highlighted by the inclusion of UHC as target 3.8 in the UN Sustainable Development Goals (SDGs).[1] According to the World Health Organisation (WHO), "UHC means that all individuals and communities receive the health services they need without suffering financial hardship".[2] This definition by the WHO is not prescriptive, but is clear in outlining that UHC does not involve providing all possible health services free of charge for everyone. Instead, UHC entails the provision of a defined package of effective services that are of sufficient quality and maximise health, equity and protection from financial hardship.[3-5]

Given the resource constraints that countries face, particularly low- and middle-income countries, challenging decisions will need to be made on what services will be provided free of charge and which services will not.[6-7] When working toward UHC, governments will therefore have to define a health benefits package that is free at the point of use, sometimes referred to as a basic or essential package of health services. Health financing reforms, and how best to implement selected services to maximise quality and access, will also need to be considered.[6-10] Best practice in priority setting commonly involves a transparent, systematic and evidence-based approach with high levels of stakeholder involvement.[6,11] Due to financial and human resource constraints, countries seeking to embark on these complex health system reforms will require administrative and analytic support for evidence-based decision making.[6,12-13]

Modelling tools can help support national analytical capacity and facilitate evidence-based priority setting.[6,14-15] Allocative efficiency analyses may be especially useful in this context as they are able to estimate and compare the health impact of various packages of services, with different budgets, while considering trade-offs between desired objectives – for example, minimising disease incidence or mortality.[16-17]

To date, allocative efficiency analyses have commonly been used to inform decision making for national disease control programmes, such as National TB or HIV Programmes.[15,18-19] There is largely unexplored potential for allocative efficiency analyses to inform priority-setting across diseases, while considering the costs, effectiveness, equity impact and financial risk protection of health benefits packages. The literature on how to consider costs and effectiveness is well established, but there is less research on the inclusion of equity measures,[20-22] and to our knowledge, financial risk protection has not yet been incorporated into allocative efficiency modelling analyses.

Rationale

Links between poverty and health have been studied extensively, highlighting the importance of socio-economic status and income inequality as determinants of health.[23-25] Likewise, indebtedness due to medical expenses is one of the main pathways into and a major cause of remaining trapped in poverty.[26-28] For individuals or households,

For peer review only

particularly those with low income, medical expenses due to health shocks can put at risk or negatively impact non-medical consumption causing financial strain or hardship.[29-30] Financial risk protection can therefore be understood as protection against the latter by the partial or full subsidisation of healthcare costs through mechanisms such as public financing and formal, informal or self-provided insurance.[31] Several measures of financial risk protection currently exist but the most frequently used indicators, adopted both by the WHO and World Bank, are measures of financial hardship and can be categorised into catastrophic health expenditure and impoverishment. Catastrophic health expenditure occurs when the proportion of a household's medical expenses relative to its income or capacity to pay exceeds a defined threshold.[32-34] Measures of impoverishment on the other hand, aim to capture the number of households pushed into, or deeper into, poverty due to healthcare costs.[34-35]

It is estimated that 808 million people incurred catastrophic health expenditures in 2010.[36] Financial risk protection to avert catastrophic health expenditure and medical impoverishment is a core component of UHC. It is explicitly referred to in target 3.8 of the SDGs, but countries are not on track to meet targets for financial risk protection.[37] When defining or updating national health benefits packages, governments should therefore consider financial risk protection as a key dimension of the priority setting process alongside the maximisation of health benefits. If allocative efficiency and modelling analyses are to inform policies for UHC and the contents of health benefits packages, a method to estimate financial risk protection must be incorporated into the analyses. A scoping review of financial risk protection measurement methods, and how they could be applied to allocative efficiency analyses, can provide a foundation for modelling efforts to become more relevant in light of the ongoing push toward UHC. No such review has yet been conducted and this protocol proposes to address this gap.

Objectives

The primary objective of the proposed scoping review is to outline the different methods used to measure financial risk protection, and discuss their strengths and weaknesses as well as application to specific services or health packages. Subsequently, the review will seek to consider how existing financial risk protection measurement methods could be applied to allocative efficiency analyses of:

1. Disease-specific health packages
2. Health benefits packages that address multiple diseases

The secondary objective of the review will be to build on the work carried out by the World Bank and WHO to track progress toward UHC and financial risk protection.[38-41] The review will compare the amount of research on financial risk protection by country and region against the burden of financial hardship to establish any mismatch between research focus and burden.

METHODS AND ANALYSIS

Scoping reviews benefit from a breadth of content by providing an overview of all the research in a given subject area. They also achieve depth through the mapping and

For peer review only

interpretation of research.[42] The objectives of the proposed review lend themselves well to such an approach. They are broad in their scope, but precise in their aim to map existing research on financial risk protection measurement methods while considering how they could be applied to allocative efficiency analyses. The proposed scoping review will follow the six stages outlined in the framework by Arksey and O'Malley,[42] hereinafter referred to as Arksey and O'Malley's framework, which are discussed in detail below. The recommendations by Levac et al.,[43] who further clarify and build on Arksey and O'Malley's framework, will also be incorporated throughout the review process. The reporting of the review will comply with the PRISMA checklist recently developed for scoping reviews.[44]

Stage 1: Identifying the research questions

The overarching question of the proposed review will guide the search strategy and the interpretation and reporting of the results: What are the different methods currently used to measure financial risk protection? This question should be broad enough to ensure that all the existing literature is captured and analysed in the review. Two sub-questions have also been identified, which will focus on the application of existing methods to health services or packages, and their potential to be included in allocative efficiency analyses. The two sub-questions are as follows: (1) How have different methods been used to estimate financial risk protection for health services or packages? (2) How could existing financial risk protection measurement methods be applied to allocative efficiency analyses? These questions will be subjected to iterative thinking as the review is carried out. While the overarching question is unlikely to change, the sub-questions proposed may be refined or additional questions included.

Stage 2: Identifying relevant studies

Stage 2 of the scoping review will involve identifying relevant studies for selection. A systematic search strategy, which is guided by the overarching question and its sub-components, will be used to carry out this stage of the review. The following electronic databases will be searched: (1) PubMed; (2) Scopus; (3) Web of Science; and (4) Google Scholar. The initial set of search terms and strategy proposed in table 1 and supplementary file 1 respectively, generates 1594 results on PubMed. The search terms and search strategy have been formulated to identify research on the measurement of financial risk protection, and how the different methods have been applied. Search results will be downloaded and imported using EndNote, and duplicates will be deleted prior to screening according to the inclusion and exclusion criteria set in Stage 3.

Table 1: List of search terms and search strategy

Search terms for financial risk protection	Search terms for methods used and their application
"Financial risk protection" OR<br>"Financial hardship " OR<br>"Financial protection" OR<br>"Financial protection in health" OR<br>"Catastrophic health expenditure" OR<br>"Catastrophic medical expenditure" OR	"Universal health coverage" OR<br>"UHC" OR<br>"Health benefits package" OR<br>"HBP" OR<br>"Basic package of health services" OR<br>"BPHS" OR

For peer review only - http://bmjopen.bmj.com/site/about/guidelines.xhtml

"Catastrophic health expenditure risk" OR<br>“Catastrophic medical expenditure risk” OR<br>“Catastrophic health payment” OR<br>“Catastrophic medical payment” OR<br>“CHE” OR<br>“CMP” OR<br>“Medical induced poverty” OR<br>“Health induced poverty” OR<br>“Payment-induced poverty” OR<br>“Catastrophic payment” OR<br>“Catastrophic cost” OR<br>"Health Impoverishment" OR<br>"Medical Impoverishment" OR<br>"Extended cost-effectiveness analysis" OR<br>"ECEA"	“Essential package of health services” OR<br>“EPHS” OR<br>“Priority setting” OR<br>“Health policy” OR<br>“Resource allocation” OR<br>“Allocative efficiency” OR<br>“Methodology” OR<br>“Measurement” OR<br>“Modelling” OR<br>“Distributional analysis” OR<br>“Tracking” OR<br>“Monitoring” OR<br>“Estimating” OR<br>“Quantifying” OR<br>“Threshold” OR<br>“Healthcare financing” OR<br>“Health insurance” OR<br>“Social health insurance” OR<br>“National health insurance”<br>“National health programs”

Relevant grey literature on financial risk protection and UHC published by institutions or organisations working on these topics will also be scanned. Examples of such institutions or organisations are the World Health Organisation (WHO), World Bank, Disease Control Priorities Network, Centre for Global Development, International Decision Support initiative (iDSI), Health Policy Plus (HP+), United Nations Department of Economic and Social Affairs (UN DESA), and the Health Intervention and Technology Assessment Program (HITAP). The reference lists of all literature deemed relevant according to the inclusion and exclusion criteria from Stage 3 will then be hand-searched. In line with Arksey and O’Malley’s framework and Levac et al., the search terms and search strategy will be improved in an iterative process as we become more familiar with the literature.

Stage 3: Selecting studies

The third stage of the review will involve setting inclusion and exclusion criteria. Preliminary inclusion and exclusion criteria are proposed below, but these will be subjected to iterative thinking as specified by Arksey and O’Malley. Studies generated by the search will then be screened according to the inclusion and exclusion criteria in a two-step selection process. The first screen will be based on the relevance of the titles and abstracts, followed by a second screen after reading the remaining articles in full.

Inclusion criteria

Peer-reviewed journal articles and grey literature measuring or discussing methodologies for measuring financial risk protection will be included. Research estimating or conceptualising financial risk protection using either quantitative or qualitative techniques,

For peer review only

regardless of the year published, geographic location, disease area of focus, health services or population groups considered will also be included.

Exclusion criteria

Papers that lack discussion either on the methodology behind the measures, their application to health services, or implications for priority setting will be excluded. Papers that cannot be accessed through institutional log-in, or in languages other than English and French will also be excluded.

Stage 4: Charting the data

The data from studies identified and selected for final inclusion in Stage 3, will be collected and charted. A data collection tool will be developed to collect key characteristics from the journal papers and grey literature included, such as the methods used, application to health services or packages, and country or region of interest. The tool will be developed in an iterative manner during the data collection process. Any decisions to amend the tool during the data collection process, and any changes to the overall review protocol will be documented and reported appropriately. Both descriptive statistics and qualitative analyses will then be carried out on the data collected before reporting the results.

Data will be aggregated and charted according to the different characteristic collected. For example, the number of studies conducted by or concerned with a given type of method, or by geographic region of interest. While there is an ongoing initiative to track progress toward UHC and financial risk protection on a global scale,[38-41] this does not map the amount of research conducted by geographic region. Therefore, using the data extracted we will attempt to compare the amount of research conducted on financial risk protection with the burden of financial hardship by geographic region. Given that the focus of the proposed review is on mapping the different methodologies available to measure financial risk protection, a thematic analysis will also be undertaken to summarise and better understand the different approaches and any overlap or links between them. The strengths, weaknesses, and the applications of different methodologies will then be discussed and investigated in the review based on the data extracted.

Stage 5: Collating, summarising and reporting the results

The data collected in Stage 4 will be collated, summarised and reported in order to effectively map the different methods currently used to measure financial risk protection. Papers on the different methods used to measure financial risk protection will be classified according to the different characteristics identified and gathered through data extraction and charting. Tables and graphical presentations such as bar charts will be used to present the data in an aggregated manner, to provide an overview of the methods and how they could be included in allocative efficiency modelling. The latter will be assessed according to certain characteristics collected for different measurement methods, such as data requirements and application to individual health services or packages.

Stage 6: Consultation

For peer review only

The consultation stage aims to involve specialists in financial risk protection and allocative efficiency modelling, as these will likely be the primary target audience of the work. The consultation stage was described as optional but desirable by Arksey and O'Malley. Since then, the recommendations outlined by Levac et al. have suggested this should be a required step as it adds methodological rigour. In line with the recommendation to establish a clear purpose for the consultation, the following two objectives are proposed due to their potential to strengthen the review. First, to ascertain whether any methods or approaches to measure financial risk protection have not been captured by the review. Second, to assess the accuracy and instructiveness of the review and the results reported.

Study timeline

The first and second stages of this study started in June 2018 and were required to develop this protocol. Stage 3 of the review commenced in October 2018 and took five months to complete by February 2019. The hand-searching of included articles and the fourth stage are expected to take four months and finish by June 2019. An additional two months have been assigned for Stages 5 and 6, and for reporting the results. The estimated completion date of this scoping review is therefore August 2019.

Patient and public involvement

Patients and the public were not involved in the development of this protocol. If specialists agree to be involved in the consultation stage, details of this will be included in the final scoping review.

DISCUSSION (ETHICS AND DISSEMINATION)

Upon completion of all the steps outlined in this protocol, the findings of the proposed scoping review will be disseminated through the submission of a paper for peer-reviewed publication and conference presentations. The findings will also be shared with the World Health Organisation, the Disease Control Priorities Network, and other organisations working to implement and estimate the impact of health benefits packages in a move toward UHC or that are involved in allocative efficiency modelling. This study does not require ethical approval as only secondary data will be used.

The proposed scoping review will be the first to comprehensively map the status-quo of research on the measurement of financial risk protection, both in terms of methodological developments and empirical work. The extraction of data from included literature is another advantage given that data extraction does not always feature in scoping reviews.[45] However, there are key limitations to the proposed review. First, the different stages have and will continue to be undertaken by the corresponding author only, under the supervision of co-authors. This introduces a greater margin for error and potential bias, particularly for stages 3 and 4.[46-47] Second, while the strengths, weaknesses, and applications of different methodologies will be discussed, the quality of included studies will not be considered as would commonly be the case in systematic reviews.[45] Third, the review will

For peer review only - http://bmjopen.bmj.com/site/about/guidelines.xhtml

fail to capture some relevant empirical work published in languages other than English or French.

The global movement toward UHC is accelerating, and as governments undertake policy reforms it is essential that the decisions made are transparent, systematic and evidence-based. Allocative efficiency analyses can inform such reforms through a systematic and evidence-based approach. However, measures of financial risk protection will need to be incorporated into allocative efficiency modelling if the efforts are to be useful and relevant to decision makers. The proposed scoping review outlined in this protocol can enable the latter by mapping the different available approaches for measuring financial risk protection, and discussing their suitability for analyses seeking to inform priority setting in health.

AUTHOR CONTRIBUTIONS

All authors contributed meaningfully to the preparation, drafting and editing of this scoping review protocol. GAJ and HHB conceived the idea and GAJ led protocol development. GAJ (corresponding author) conceptualised the research questions and core research plan details, before preparing the initial draft of this manuscript. JS and HHB provided critical input on the draft, research questions, and methodology. All authors approved the final submitted manuscript, after several iterations and rounds of editing, and agree to be accountable for all aspects of this protocol.

ACKNOWLEDGEMENTS

Not applicable.

DATA SHARING STATEMENT

There are no additional data available.

FUNDING

This work was supported by the Bill and Melinda Gates Foundation grant number OPP1179683.

COMPETING INTERESTS

All authors declare they have no competing interests.

PATIENT CONSENT

For peer review only

None required.

REFERENCES

1. United Nations. https://sustainabledevelopment.un.org/ (accessed 19 August 2018).
2. World Health Organisation. Universal Health Coverage: Fact Sheet. *World Health Organisation* 2017 http://www.who.int/news-room/fact-sheets/detail/universal-health-coverage-(uhc) (accessed 19 August 2018).
3. World Health Organisation. The world health report: health systems financing: the path to universal coverage. *Geneva: World Health Organisation* 2010.
4. World Health Organisation. Making fair choices on the path to universal health coverage. *Geneva: World Health Organisation* 2014.
5. Norheim OF. Ethical priority setting for universal health coverage: challenges in deciding upon fair distribution of health services. *BMC Med* 2016 Dec;14:75.
6. Glassman A, Giedion U, Smith PC, eds. What's in, what's out: designing benefits for universal health coverage. *Washington, D.C: Center for Global Development,* 2017
7. Jamison DT, Gelband H, Horton S, *et al.*, eds. Disease Control Priorities, (Volume 9): Improving Health and Reducing Poverty. *The World Bank* 2017
8. Peters DH, Garg A, Bloom G, *et al*. Poverty and access to health care in developing countries. *Ann N Y Acad Sci* 2008;1136:161-71.
9. Dieleman JL, Sadat N, Chang AY, *et al*. Trends in future health financing and coverage: future health spending and universal health coverage in 188 countries, 2016–40. *Lancet* 2018;391:1783-98.
10. Chalkidou K, Glassman A, Marten R, *et al*. Priority-setting for achieving universal health coverage. *Bull World Health Organ* 2016;94:462.
11. Barasa EW, Molyneux S, English M, *et al*. Setting healthcare priorities at the macro and meso levels: a framework for evaluation. *Int J Health Policy Manag* 2015;4:719.
12. Nuyens Y. Setting priorities for health research: lessons from low- and middle-income countries. *Bull World Health Organ* 2007;85:319-321.
13. Glassman A, Giedion U, Sakuma Y, *et al*. Defining a health benefits package: what are the necessary processes?. *Health Syst Reform* 2016;2:39-50.
14. Baltussen R, Niessen L. Priority setting of health interventions: the need for multi-criteria decision analysis. *Cost Eff Resour Alloc* 2006;4:14.
15. Stuart RM, Grobicki L, Haghparast-Bidgoli H, *et al.* How should HIV resources be allocated? Lessons learnt from applying Optima HIV in 23 countries. *J Int AIDS Soc* 2018;21:e25097.
16. Kerr CC, Stuart RM, Gray RT, *et al*. Optima: a model for HIV epidemic analysis, program prioritization, and resource optimization. *J Acquir Immune Defic Syndr* 2015;69:365-76.
17. Pearson R, Killedar M, Petravic J, *et al.* Optima Nutrition: an allocative efficiency tool to reduce childhood stunting by better targeting of nutrition-related interventions. *BMC Public Health* 2018;18:384.
18. Kelly SL, Martin-Hughes R, Stuart RM, *et al*. The global Optima HIV allocative efficiency model: targeting resources in efforts to end AIDS. *Lancet HIV* 2018;5:e190-8.

For peer review only - http://bmjopen.bmj.com/site/about/guidelines.xhtml

19. Stuart RM, Kerr CC, Haghparast-Bidgoli H, *et al*. Getting it right when budgets are tight: Using optimal expansion pathways to prioritize responses to concentrated and mixed HIV epidemics. *PLoS One*. 2017;12:e0185077.
20. Cleary S, Mooney G, McIntyre D. Equity and efficiency in HIV-treatment in South Africa: the contribution of mathematical programming to priority setting. *Health Econ* 2010;19:1166-80.
21. Norheim OF, Baltussen R, Johri M, *et al*. Guidance on priority setting in health care (GPS-Health): the inclusion of equity criteria not captured by cost-effectiveness analysis. *Cost Eff Resour Alloc* 2014;12:18.
22. Baltussen R, Jansen MP, Mikkelsen E, *et al*. Priority setting for universal health coverage: we need evidence-informed deliberative processes, not just more evidence on cost-effectiveness. *Int J Health Policy Manag* 2016;5:615.
23. Adler NE, Ostrove JM. Socioeconomic status and health: what we know and what we don't. *Ann N Y Acad Sci* 1999;1;896(1):3-15.
24. Marmot M. Status syndrome: How your social standing directly affects your health. *A&C Black* 2005.
25. Pickett KE, Wilkinson RG. Income inequality and health: a causal review. *Soc Sci Med* 2015;1;128:316-26.
26. van Damme W. Out-of-pocket health expenditure and debt in poor households: evidence from Cambodia. *Trop Med Int Health* 2004;9(2):273–280.
27. Krishna A. Pathways out of and into poverty in 36 villages of Andhra Pradesh, India. *World Dev* 2006;34(2):271–288.
28. Sen B. Drivers of escape and descent: changing household fortunes in rural Bangladesh. *World Dev* 2003;1;31(3):513-34.
29. Russell S. Ability to pay for health care: concepts and evidence. *Health Policy and Plan* 1996;11:219–237
30. Wagstaff A. The economic consequences of health shocks: evidence from Vietnam. *J Health Econ* 2007;26(1):82-100.
31. O'Donnell O. Financial Protection Against Medical Expense. Tinbergen Institute Discussion Papers. *Tinbergen Institute* 2019;10.
32. Berki SE. A look at catastrophic medical expenses and the poor. *Health Aff* 1986;5(4):138-45.
33. Xu K, Evans DB, Kawabata K, Zeramdini R, Klavus J, Murray CJ. Household catastrophic health expenditure: a multicountry analysis. *Lancet* 2003;362(9378):111-7
34. Wagstaff A, Doorslaer EV. Catastrophe and impoverishment in paying for health care: with applications to Vietnam 1993–1998. *Health Econ* 2003;12(11):921-33.
35. Van Doorslaer E, O'Donnell O, Rannan-Eliya RP, Somanathan A, Adhikari SR, Garg CC, Harbianto D, Herrin AN, Huq MN, Ibragimova S, Karan A. Effect of payments for health care on poverty estimates in 11 countries in Asia: an analysis of household survey data. *Lancet* 2006 Oct 14;368(9544):1357-64.
36. Wagstaff A, Flores G, Hsu J, *et al*. Progress on catastrophic health spending in 133 countries: a retrospective observational study. *Lancet Glob Health* 2018;6:e169-79.
37. Verguet S, Woldemariam AT, Durrett WN, *et al*. Is the sustainable development goal target for financial risk protection in health realistic?. *BMJ Glob Health* 2017;2:e000216.

For peer review only

38. World Health Organisation. Global Health Observatory. http://apps.who.int/gho/portal/uhc-fp-cabinet-wrapper-v2.jsp?id=1020201 (accessed 19 August 2018). (accessed 19 August 2018).
39. Boerma T, Eozenou P, Evans D, *et al*. Monitoring progress towards universal health coverage at country and global levels. *PLoS Med* 2014;11:e1001731.
40. World Health Organization, World Bank Group. Monitoring progress towards universal health coverage at country and global levels: framework, measures and targets. *Geneva, Washington DC: World Health Organization, World Bank Group* 2014.
41. World Health Organization, World Bank Group. Tracking universal health coverage: 2017 global monitoring report. *Geneva, Washington DC: World Health Organization, World Bank Group* 2017.
42. Arksey H, O'Malley L. Scoping studies: towards a methodological framework. *Int J Soc Res Methodol* 2005;8:19-32.
43. Levac D, Colquhoun H, O'Brien KK. Scoping studies: advancing the methodology. *Implement Sci* 2010;5:69.
44. Tricco AC, Lillie E, Zarin W, O'Brien KK, Colquhoun H, Levac D, Moher D, Peters MD, Horsley T, Weeks L, Hempel S. PRISMA extension for scoping reviews (PRISMA-ScR): checklist and explanation. *Ann Intern Med* 2018;169(7):467-73.
45. Armstrong R, Hall BJ, Doyle J, Waters E. 'Scoping the scope'of a cochrane review. *J Public Health* 2011;33(1):147-50.
46. Edwards P, Clarke M, DiGuiseppi C, Pratap S, Roberts I, Wentz R. Identification of randomized controlled trials in systematic reviews: accuracy and reliability of screening records. *Stat Med* 2002;21(11):1635-40.
47. Buscemi N, Hartling L, Vandermeer B, Tjosvold L, Klassen TP. Single data extraction generated more errors than double data extraction in systematic reviews. *J Clin Epidemiol* 2006;59(7):697-703.

For peer review only

Supplementary file 1: PubMed search strategy

Financial risk protection: ("Financial risk protection" OR "Financial hardship " OR “Financial protection” OR “Financial protection in health” OR "Catastrophic health expenditure" OR “Catastrophic medical expenditure” OR "Catastrophic health expenditure risk" OR “Catastrophic medical expenditure risk” OR “Catastrophic health payment” OR “Catastrophic medical payment” OR “CHE” OR “CMP” OR “Medical induced poverty” OR “Health induced poverty” OR “Payment-induced poverty” OR “Catastrophic payment” OR "Catastrophic cost" OR "Health Impoverishment" OR "Medical Impoverishment" OR "Extended cost-effectiveness analysis" OR "ECEA")

AND

Methods used and their application: (“Universal health coverage” OR “UHC” OR “Health benefits package” OR “HBP” OR “Basic package of health services” OR “BPHS” OR “Essential package of health services” OR “EPHS” OR “Priority setting” OR “Health policy” OR “Resource allocation” OR “Allocative efficiency” OR “Methodology” OR “Measurement” OR “Modelling” OR “Distributional analysis” OR “Tracking” OR “Monitoring” OR “Estimating” OR “Quantifying” OR “Threshold” OR “Health financing” OR “Health insurance” OR “Social health insurance” OR “National health insurance” OR “National health programs”)

AND

Additional Limits: Humans[Mesh]

For peer review only
